# Interpretable Machine Learning for Predicting Early Onset Hypertension in the US

**DOI:** 10.5888/pcd23.260109

**Published:** 2026-10-22

**Authors:** Li-Wei Wu, Kiyoung Kim, Peter Washington, Chanhyun Park

**Affiliations:** 1 Health Outcomes Division, College of Pharmacy, The University of Texas at Austin, Austin, Texas; 2 Department of Epidemiology and Biostatistics, School of Public Health, Texas A&M University, College Station, Texas; 3 Division of Clinical Informatics and Digital Transformation, Department of Medicine, University of California, San Francisco

## Abstract

**Introduction:**

Early onset hypertension is often overlooked despite its association with lifelong cardiovascular disease risk. We developed an interpretable machine learning model to predict early onset hypertension in US adults using the 2022 Medical Expenditure Panel Survey, incorporating demographic, socioeconomic, health care utilization, and comorbidity features.

**Methods:**

We analyzed data on adults aged 55 years or younger. Hypertension was defined by *International Classification of Diseases, 10th Revision, Clinical Modification* code number I10 or self-reported physician diagnosis at or before age 55 years. Eighty-four variables were screened; Least Absolute Shrinkage and Selection Operator reduced the set to 45 variables. Extreme gradient boosting was trained with stratified 5-fold cross-validation. Performance metrics included accuracy, precision, recall, F2 score, area under the receiver operating characteristic curve (ROC AUC), and area under the precision-recall curve (PR AUC). We assessed fairness across subgroups and used Shapley Additive Explanations to quantify feature influence.

**Results:**

Among 9,850 adults, 1,647 (weighted prevalence, 15.2%) had hypertension. Discrimination was strong, with a ROC AUC of 0.807 (95% CI, 0.787–0.827), PR AUC of 0.486 (95% CI, 0.439–0.530), recall of 0.848 (95% CI, 0.816–0.878), and F2 score of 0.606 (95% CI, 0.578–0.634). Key predictors included older age, high body mass index, and a diagnosis of high cholesterol. Fairness analyses showed slightly higher true positive rates for Hispanic (+0.11) and Asian (+0.08) people, with performance for African American people modestly lower than that for White people.

**Conclusion:**

Both medical and nonmedical predictors contributed meaningfully, with high cholesterol particularly influential among adults older than 40 years and race and ethnicity especially important among African American adults. Prospective validation and routine fairness audits are warranted to ensure equitable benefit across populations. This interpretable model supports recall-oriented screening to identify younger adults at risk of hypertension for further evaluation and early intervention.

**Summary**

**What is known on this topic?**

Early onset hypertension is often underrecognized despite its lifelong cardiovascular disease risks. Existing prediction models primarily assess older adults, infrequently incorporate both medical and nonmedical predictors, and rarely evaluate algorithmic fairness.

**What is added by this report?**

We developed an interpretable model with strong discrimination and identified age, body mass index, and high cholesterol as key predictors. Subgroup analyses indicated differential feature importance and performance, underscoring the need for ongoing fairness evaluation.

**What are the implications for public health practice?**

By prioritizing recall-oriented screening, the model may support earlier identification of adults at risk of hypertension and facilitate timely lifestyle intervention, monitoring, and equitable prevention strategies.

## Introduction

Early onset hypertension is generally defined as the development of hypertension at or before the age of 55, although some studies use younger cutoffs (eg, 45 y) (1). In the US, approximately 20% of adults younger than 40 have hypertension (2). Although prevalence is lower than in older adults, younger adults often have low levels of awareness of the condition and suboptimal disease control (3). Monitoring hypertension in younger people is crucial, as prolonged exposure to hypertension can significantly increase the risk of cardiovascular, cerebrovascular, and renal comorbidities over a lifetime (4,5). People diagnosed with hypertension before age 35 years have significantly increased odds of structural cardiovascular abnormalities, including a 2.29-fold higher likelihood of left ventricular hypertrophy and a 2.94-fold higher likelihood of coronary calcification, compared with normotensive people. No such associations are observed among those diagnosed at age 45 or older (6). Earlier onset is also associated with higher rates of cardiovascular illness and death; hypertension diagnosed before age 45 years confers a 2.26-fold increased risk of cardiovascular disease (CVD) and a 2.59-fold increased risk of all-cause mortality relative to normotensive people (7).

Early onset hypertension is influenced by a combination of medical and nonmedical factors. Medical factors include diabetes, dyslipidemia, a family history of hypertension, obesity, and related cardiometabolic conditions (8–10). For example, people with 1 parent who has early onset hypertension have nearly double the risk, while those with both parents affected have a risk more than 3.5 times higher (11). Nonmedical factors include African American race, low socioeconomic status, high-sodium diet, physical inactivity, and limited access to health care (8,12). Educational attainment also matters; the prevalence of hypertension among college graduates is approximately 8.5 percentage points lower than the prevalence among those with a high school education or less (38.5% vs 47.0%) (2).

Although both medical and nonmedical predictors of hypertension have been identified, evidence for integrating them into a comprehensive prediction model is limited. Incorporating both domains may yield more complete and clinically useful risk stratification. Research focusing on early onset hypertension is also scarce; most literature concentrates on middle-aged or older populations. Furthermore, few studies have evaluated the algorithmic fairness of hypertension prediction models, such as subgroup differences in error rates or calibration, even though fairness is essential to prevent biased decision-making and ensure equitable access to hypertension care. To address this gap, our objective was to develop an interpretable machine learning (ML) model that predicts early onset hypertension in US adults by using a comprehensive set of medical and nonmedical predictors. Specifically, we assessed subgroup fairness across various demographic factors. In addition, we identified the most influential predictors and their direction of association. The application of ML is particularly suitable because it can effectively capture nonlinear relationships and interactions among multiple correlated variables without imposing stringent distributional assumptions (13). Conducting a fairness assessment is essential to ascertain whether predictive errors vary across demographic groups and to promote equitable population health applications. We used Shapley Additive Explanations (SHAP) to deliver transparent feature attributions at both the individual and aggregate levels, thereby linking predictions to actionable risk factors. Taken together, this approach enables a comprehensive and equitable assessment of early onset hypertension risk, providing insights that could help create tailored prevention strategies and inform clinical decision-making.

## Methods

### Data source

We used data from the 2022 Medical Expenditure Panel Survey (MEPS), which is a nationally representative panel survey of the US civilian noninstitutionalized population managed by the Agency for Healthcare Research and Quality (14). This database contains detailed medical and nonmedical information, such as patient demographic characteristics, health conditions, health status, and health care use. We used the full-year consolidated data file to obtain person-level information, linking it with the medical conditions file to access diagnosis codes for patients with early onset hypertension. The data files are publicly accessible. The institutional review board at The University of Texas at Austin determined that this study qualified for exemption from review.

In this retrospective population-based study, we included adults aged 55 years or younger. People were divided into 2 groups based on the presence of early onset hypertension (yes or no).

### Measures

Our outcome of interest was early onset hypertension, defined using *International Classification of Diseases, 10th Revision, Clinical Modification* code number I10 or self-reported physician diagnosis occurring at or before the age of 55 years. Initially, 84 medical and nonmedical variables were considered as potential predictors. These variables included demographic characteristics (eg, age, sex, race and ethnicity), socioeconomic characteristics (eg, educational level, employment status, family income), number of health care access points (eg, emergency department visits, inpatient visits), comorbidities (eg, diabetes, high cholesterol, heart disease), health status (eg, perceived physical and mental health status), behavioral factors (eg, exercise frequency, alcohol consumption, tobacco use), and history of COVID-19. A comprehensive description of these variables is provided in Supplementary Table 1 (https://doi.org/10.6084/m9.figshare.33365892).

### Statistical analysis

We compared descriptive statistics between people with and without early onset hypertension by using the Wilcoxon rank-sum test for nonnormally distributed continuous variables (emergency department visits and hospitalizations), the Welch *t* test for normally distributed continuous variables (age and body mass index [BMI], calculated as weight divided by height in meters squared), and the Pearson χ^2^ test for categorical variables. All tests were 2-sided (*P* < .05) and conducted by using unweighted data. Survey weights were applied to produce nationally representative estimates for all reported descriptive statistics and were incorporated into the development of the ML model to ensure that the model reflected the distribution of the US target population rather than the survey sample. The statistical analysis was performed by using Python version 3.13.2 (Python Software Foundation).

### ML analysis

#### Data preprocessing

The data set was randomly divided into training (70%) and testing (30%) sets, stratified by the outcome to preserve the proportion of people with early onset hypertension. Using the Least Absolute Shrinkage and Selection Operator (LASSO) with 10-fold cross-validation for feature selection, 45 of 84 predictors were retained for inclusion in the final model (15). To minimize bias caused by missing data (ie, approximately 50% missing for frequency of trouble sleeping, moderate exercise, alcohol consumption, and tobacco use), we applied the k-nearest neighbors (KNN) imputation method. The KNN imputation was fitted solely on the training data set, and the learned imputation parameters were then applied to the test data set to prevent data leakage. This approach estimates missing values by leveraging patterns among similar observations, using the mean or median of the k-nearest neighbors (16).

#### ML algorithm

We implemented the extreme gradient boosting (XGBoost) algorithm with k-fold cross-validation to ensure a reliable estimate of the model’s performance and to minimize the risk of overfitting (17). XGBoost is an ensemble classifier that sequentially trains weak classifiers to correct the errors made by previous iterations (18). We conducted hyperparameter tuning for the XGBoost classifier using a grid search with 5-fold cross-validation on the training set. The hyperparameters explored included the number of trees (ie, 200, 300, or 400), maximum tree depth (ie, 3, 4, or 5), learning rate (ie, 0.03, 0.05, 0.07, or 0.1), subsample ratio of training instances (ie, 0.7, 0.8, or 0.9), subsample ratio of columns when constructing each tree (ie, 0.7, 0.8, or 0.9), L1 regularization term (ie, 0, 0.01, or 0.1), and L2 regularization term (ie, 1, 1.5, or 2). The optimal classification threshold was then determined using 5-fold cross-validation on the training set to maximize the F2 score, emphasizing recall to better identify people with early onset hypertension. To evaluate overfitting behavior, we retrained the final XGBoost model using an internal validation set (20% of training data, stratified), applying early stopping when validation log-loss showed no improvement for 20 consecutive boosting rounds. Training and validation log-loss curves were plotted across iterations to assess convergence and overfitting. To further evaluate model stability and generalizability, we also assessed the final XGBoost model with optimized hyperparameters using 10-fold stratified cross-validation on the training set.

#### Evaluation of model performance and fairness

Model performance was evaluated by using various metrics, including accuracy, precision, recall, F2 score, the area under the receiver-operating-characteristic curve (ROC AUC), and the area under the precision-recall curve (PR AUC) (19). Because ROC AUC is insensitive to class imbalance while PR AUC is sensitive, both metrics were reported to provide a more comprehensive assessment of model performance (20). We presented the mean and 95% CI of these metrics. To measure model fairness across demographic subgroups, we analyzed the differences in true-positive rates (TPRs), false-positive rates (FPRs), false-negative rates, and the average of the TPR and FPR differences. Larger discrepancies in these metrics across subgroups indicate that the model’s performance is less equitable among groups (21).

#### Evaluation of feature importance

We used Shapley Additive Explanations (SHAP) to identify the most influential predictors in the overall population and within subgroups (22). The SHAP value indicates both the magnitude and direction of a feature’s contribution to the predicted outcome. To visualize the results, we created both a SHAP feature importance plot and a summary plot.

#### Sensitivity analysis

We conducted 4 sensitivity analyses to evaluate the robustness of model performance and feature importance. First, we implemented a neural network classifier to replace the XGBoost algorithm and compare the performance metrics of both models (23); survey weights were not applied in this sensitivity analysis due to the algorithmic constraints of neural network classifiers. Second, to ensure that the findings remained consistent across varying clinical definitions, we restricted our cohort to include people aged 18 to 40 years and redefined early onset hypertension as either a diagnosis code or self-reported physician diagnosis occurring at or before the age of 40 years. Third, we redefined early onset hypertension by combining strictly self-reported information with diagnosis codes to minimize potential recall bias. Finally, we conducted a complete-case analysis by excluding people with missing data on any variable to verify the impact of our imputation strategy. We then applied SHAP to identify the most important features within each population. All the ML models were built by using Python (version 3.13.2; Python Software Foundation), along with several Python modules, including NumPy, Pandas, Matplotlib, Scikit-learn, XGBoost, SHAP, and AIF360.

## Results

### Population characteristics

A total of 9,850 adults aged 18 to 55 years were included in the study, representing an estimated 163.4 million US civilians in this age group. Among them, 1,647 people (unweighted prevalence, 16.7%; weighted prevalence, 15.2%) were identified as having early onset hypertension (Supplementary Figure 1). Compared with people without early onset hypertension, those with this condition were older (weighted mean age, 44.0 y vs 34.8 y), had a higher BMI (32.6 vs 27.7), had a higher weighted prevalence of high cholesterol (42.2% vs 11.2%) and diabetes (18.5% vs 3.0%), and were more likely to be African American (16.9% vs 12.5%) (Supplementary Table 2).

### Model performance

The optimal hyperparameter combination selected for the final XGBoost model included 200 trees, a maximum tree depth of 5, a learning rate of 0.03, a subsample ratio of 0.9 for training instances, a subsample ratio of 0.7 for columns when constructing each tree, an L1 regularization term of 0, and an L2 regularization term of 1. Training and validation log-loss both converged without divergence, with the validation log-loss stabilizing after approximately 50 rounds. Early stopping was triggered at iteration 173, at which point the best validation log-loss was 0.3380, confirming that additional boosting rounds did not improve generalization. Although training log-loss continued to decrease across boosting rounds, the validation log-loss stabilized and showed no upward trend, indicating that the model did not overfit the training data (Supplementary Figure 2). Ten-fold stratified cross-validation on the training set yielded a mean ROC AUC of 0.823 (95% CI, 0.812–0.834) and PR AUC of 0.491 (95% CI, 0.462–0.517), with low variance across folds, confirming model stability and generalizability (Supplementary Table 3). The confusion matrix on the test set (n = 2,955) demonstrated 419 true positives, 75 false negatives, 1,401 true negatives, and 1,060 false positives, reflecting the model’s emphasis on maximizing recall for early onset hypertension identification (Supplementary Figure 3). The XGBoost model demonstrated strong discriminative performance on the test set, with an average ROC AUC of 0.807 (95% CI, 0.787–0.827) and PR AUC of 0.486 (95% CI, 0.439–0.530) (Table). Overall performance metrics included an accuracy of 0.616 (95% CI, 0.598–0.632), a precision of 0.283 (95% CI, 0.260–0.305), a recall of 0.848 (95% CI, 0.816–0.878), and an F2 score of 0.606 (95% CI, 0.578–0.634). The neural network algorithm exhibited comparable predictive performance with ROC AUC of 0.812 (95% CI, 0.792–0.833) and PR AUC of 0.482 (95% CI, 0.435–0.527). The sensitivity analysis restricting the cohort to adults aged 40 years or younger yielded an ROC AUC of 0.804 (95% CI, 0.769–0.838) and PR AUC of 0.320 (95% CI, 0.249–0.397). When early onset hypertension was redefined using both self-reported information and diagnosis codes, the model achieved an ROC AUC of 0.826 (95% CI, 0.803–0.847) and PR AUC of 0.323 (95% CI, 0.276–0.383). The complete-case analysis excluding people with missing data produced an ROC AUC of 0.803 (95% CI, 0.765–0.839) and PR AUC of 0.468 (95% CI, 0.383–0.556). Across all sensitivity analyses, model performance remained broadly consistent with the primary analysis, supporting the robustness of the results (Supplementary Table 4).

**Table:** Overall Performance Metrics of the Evaluated Machine Learning Models, Medical Expenditure Panel Survey, 2022^a^

Analysis type	ROC AUC	PR AUC	Accuracy	Precision	Recall	F2 score
Primary						
XGBoost	0.807 (0.787–0.827)	0.486 (0.439–0.530)	0.616 (0.598–0.632)	0.283 (0.260–0.305)	0.848 (0.816–0.878)	0.606 (0.578–0.634)
Sensitivity						
Neural network	0.812 (0.792–0.833)	0.482 (0.435–0.527)	0.680 (0.664–0.696)	0.320 (0.293–0.344)	0.814 (0.778–0.847)	0.622 (0.593–0.651)

Abbreviations: PR AUC, area under the precision-recall curve; ROC AUC, area under the receiver-operator-characteristic curve; XGBoost, eXtreme Gradient Boosting.

^a^ Data are presented as mean and 95% CI.

### Model fairness

Fairness assessments indicated potential predictive bias across some subgroups (Figure 1 and Supplementary Figure 4). For example, true-positive rates were slightly higher for Hispanic (+0.11) and Asian (+0.08) people, while performance for African American people was modestly lower than that for White people. Additionally, people who consumed alcohol more than 3 times per week demonstrated lower true-positive rates compared with nondrinkers. Overall, people who were older, had higher BMI, had other chronic conditions, reported poor or fair physical health, or experienced sleep difficulties were less likely to be predicted as positive.

**Figure 1:**
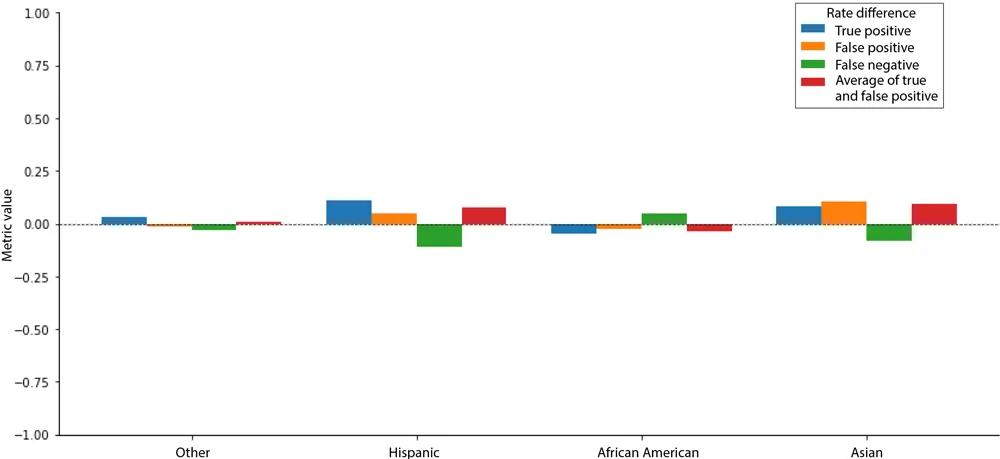
Fairness assessment across racial and ethnic groups, machine learning model analysis for development of early onset hypertension, Medical Expenditure Panel Survey, 2022. White race is the reference group.

**Table:** 

Rate difference	Race and ethnicity, metric value			
	Other	Hispanic	African American	Asian
True positive	0.03	0.11	−0.05	0.08
False positive	−0.01	0.05	−0.03	0.11
False negative	−0.03	−0.11	0.05	−0.08
Average of true positive and false positive	0.01	0.08	−0.04	0.09

### Feature importance

A SHAP feature importance and summary plot highlighted the top 10 features contributing to the prediction of early onset hypertension. These included older age, elevated BMI, a diagnosis of high cholesterol, poorer perceived health status, male sex, non-Hispanic ethnicity, living in the South, a higher number of emergency department visits, and diabetes diagnosis (Figure 2). In the sensitivity analyses where early onset hypertension was redefined as occurring at age 40 years or younger, defined using both self-reported and diagnosis codes, or restricted the analysis to people without missing data, nearly all key predictors remained consistent, with age and BMI remaining the most influential (Supplementary Figures 5–7).

**Figure 2:**
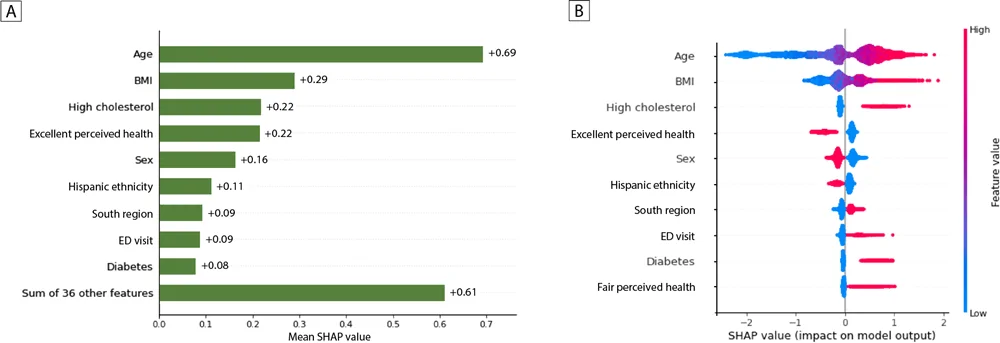
Importance and summary plots of the top 10 SHAP features from the XGBoost model, machine learning model analysis for development of early onset hypertension, Medical Expenditure Panel Survey, 2022. A, SHAP features importance plot; B, SHAP summary plot. Abbreviations: BMI, body mass index; ED, emergency department; SHAP, Shapley Additive Explanations.

**Table:** 

SHAP feature	Mean SHAP value
Age	+0.69
BMI	+0.29
High cholesterol	+0.22
Excellent perceived health	+0.22
Sex	+0.16
Hispanic ethnicity	+0.11
South region	+0.09
ED visit	+0.09
Diabetes	+0.08
Sum of 36 other features	+0.61

Some features showed greater importance for some subgroups in predicting early onset hypertension (Figure 3 and Supplementary Figure 8). For example, among people older than 40 years, a diagnosis of high cholesterol (mean SHAP value, ~0.3) had greater influence than in those aged 40 years or younger. In addition, for African American people, race (being African American) was a more significant predictor, while sex was less influential. Furthermore, among people who had coronary heart disease or had experienced a heart attack, a diagnosis of high cholesterol and a diabetes diagnosis had a stronger impact than in those without these conditions.

**Figure 3:**
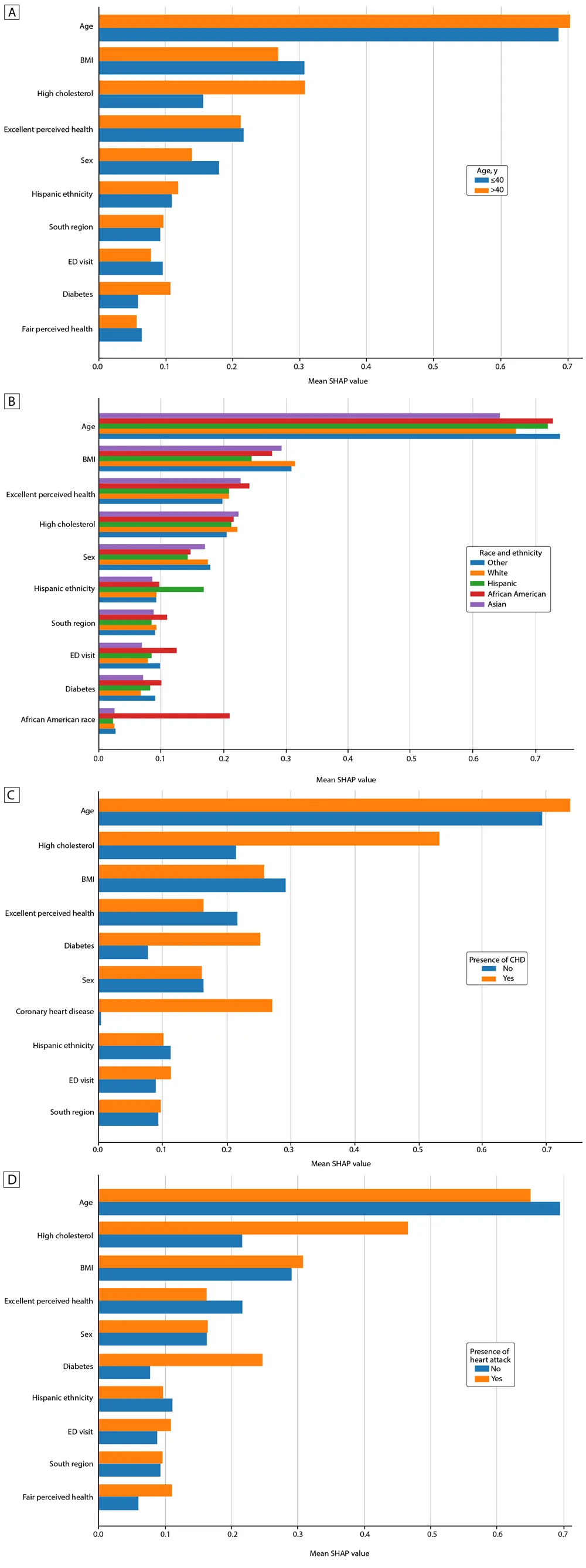
Importance of SHAP features in machine learning model analysis for development of early onset hypertension, Medical Expenditure Panel Survey, 2022. A, age; B, race and ethnicity; C, CHD; and D, heart attack. Abbreviations: BMI, body mass index; CHD, coronary heart disease; ED, emergency department; SHAP, Shapley Additive Explanations.

**Table:** 

SHAP feature	Age (graph A)	
	≤40 years	>40 years
Age	0.69	0.70
BMI	0.31	0.27
High cholesterol	0.16	0.31
Excellent perceived health	0.22	0.21
Sex	0.18	0.14
Hispanic ethnicity	0.11	0.12
South region	0.09	0.10
ED visit	0.10	0.08
Diabetes	0.06	0.11
Fair perceived health	0.06	0.06

**Table:** 

SHAP feature	Race and ethnicity (graph B)				
	Other	White	Hispanic	African American	Asian
Age	0.74	0.67	0.72	0.73	0.65
BMI	0.31	0.32	0.25	0.28	0.30
Excellent perceived health	0.20	0.21	0.21	0.24	0.23
High cholesterol	0.21	0.22	0.21	0.22	0.23
Sex	0.18	0.18	0.14	0.15	0.17
Hispanic ethnicity	0.09	0.09	0.17	0.10	0.09
South region	0.09	0.09	0.09	0.11	0.09
ED visit	0.10	0.08	0.09	0.13	0.07
Diabetes	0.09	0.07	0.08	0.10	0.07
African American race	0.03	0.03	0.03	0.21	0.03

**Table:** 

SHAP feature	CHD (graph C)	
	No	Yes
Age	0.69	0.74
High cholesterol	0.22	0.53
BMI	0.29	0.26
Excellent perceived health	0.22	0.16
Diabetes	0.08	0.25
Sex	0.16	0.16
CHD	0.00	0.27
Hispanic ethnicity	0.11	0.10
ED visit	0.09	0.11
South region	0.09	0.10

**Table:** 

SHAP feature	Heart attack (graph D)	
	No	Yes
Age	0.69	0.65
High cholesterol	0.22	0.46
BMI	0.29	0.31
Excellent perceived health	0.22	0.16
Sex	0.16	0.17
Diabetes	0.08	0.25
Hispanic ethnicity	0.11	0.10
ED visit	0.09	0.11
South region	0.09	0.10
Fair perceived health	0.06	0.11

## Discussion

This US population-based study applied an ML model that demonstrated strong discrimination (ROC AUC = 0.807; PR AUC = 0.486) for predicting the presence of hypertension among adults aged 55 years or younger. However, our findings showed differences in subgroup performance that may reflect potential fairness concerns. Specifically, Hispanic and Asian people, younger adults, and those with lower BMI were more likely to be predicted as having early onset hypertension. In contrast, people who are frequent alcohol consumers, have other comorbidities, self-report poor physical health, or experience trouble sleeping were less likely to be predicted as having early onset hypertension. The main features driving the model’s prediction of early onset hypertension were older age and elevated BMI. These findings are consistent with established hypertension epidemiology, such as age-related arterial stiffness and adiposity, and several features had greater influence in some subgroups (24,25).

Our model’s leading predictors (age, BMI, dyslipidemia) align with prior ML and epidemiologic studies. Similar to our findings, various studies that applied ML algorithms to predict the risk of hypertension have reported comparable discrimination and overlapping risk factors. One study conducted in Norway that aimed to predict hypertension risk over an 11-year period used ML models such as XGBoost, elastic regression, KNN, support vector machines (SVM), and random forest. The ROC AUC ranged from 0.75 to 0.78. The researchers identified age, systolic and diastolic blood pressure, and BMI as the most influential predictors (26). Another study employing the XGBoost model focused on predicting a 1-year risk of hypertension and found that type 2 diabetes, lipid disorders, CVDs, and mental illness were the key driving factors, achieving an ROC AUC of 0.87 (27). Research from Japan applied XGBoost, an ensemble model, and logistic regression, resulting in ROC AUC scores from 0.859 to 0.881. In this study, systolic and diastolic blood pressure, BMI, and age were identified as key predictors using the XGBoost algorithm (28). In another investigation that used logistic regression, random forest, Naïve Bayes, XGBoost, and artificial neural networks, the AUC values ranged from 0.765 to 0.825, with critical predictors including blood pressure, fatty liver index, and age (29). Focusing on early onset hypertension, a study from Mexico examined nonmedical information and laboratory data as potential predictors. Researchers applied XGBoost, logistic regression, and SVM models and reported that that sleep disorders, a sedentary lifestyle, the consumption of high-fat foods, and the consumption of energy drinks were influential factors for young and relatively healthy adults (30). These findings indicate that the key drivers of hypertension span both medical and nonmedical domains. Collectively, these studies reinforce that both clinical and nonmedical drivers matter. In terms of discriminative performance, our XGBoost model achieved an ROC AUC of 0.807 (95% CI, 0.787–0.827), broadly comparable to existing ML-based hypertension prediction models. Unlike prior models that incorporated laboratory values, our model relied primarily on survey-based variables, which likely explains the modest performance gap relative to higher-performing models. Beyond discrimination, our study offers 2 contributions largely absent from prior work: subgroup-specific feature importance analysis across demographic, socioeconomic, and clinical characteristics, and a systematic fairness evaluation quantifying differential model performance across subgroups, both of which are critical for ensuring the equitable application of ML-based screening tools in diverse populations. Our strong predictive performance supports recall-oriented, population-level screening workflows. The emphasis should be on maximizing recall to identify people at risk for further evaluation, rather than making conclusive clinical diagnoses. In practice, positive screens would prompt standardized blood pressure measurement including out-of-office monitoring when appropriate, lifestyle counseling, such as dietary sodium reduction, weight management, physical activity, and tailored follow-up (31).

That some factors had greater influence in some subgroups is noteworthy. We found that among older people (>40 y), a diagnosis of high cholesterol had a greater influence on predicting early onset hypertension than it did among younger people. For people with coronary heart disease or for those who had experienced a heart attack, diagnoses of high cholesterol and diabetes were especially influential. A potential reason might be the natural decline in cardiovascular health associated with aging or disease, making the effect of these factors on hypertension more pronounced. Moreover, our findings suggest that race is a particularly strong predictor for African Americans. A similar study showed that African American people have a higher risk for early onset hypertension, with odds ratios of 5.08 for people diagnosed before the age of 35 and 2.28 for those aged 45 years or older (9). Additionally, another study found that, on average, hypertension is diagnosed 5 years earlier in African American adults compared with White adults (32). These disparities likely reflect structural and environmental exposures (eg, neighborhood disadvantage, chronic stress, access to care) rather than inherent biologic differences.

We found that hypertension predictions were biased against some groups. Hispanic and Asian people, younger adults, and those with lower BMI were more often predicted to have early onset hypertension. In contrast, regular alcohol consumers, those with comorbidities, and people with poor physical health or sleep issues were less likely to be predicted to have early onset hypertension. Fairness is critical because systematic differences in error rates (eg, TPRs, FPRs) can entrench inequities in case-finding. However, this topic has not been extensively addressed in previous hypertension research. The observed biases in predictions may arise from the inadequate representation of some demographic groups within the data (33). For instance, our study specified a younger cohort, with the prevalence of CVD being less than 5%. Consequently, patients with CVD may have generated proportionately fewer data compared with people without these conditions. When the ML model is trained on these data, it can lead to underrepresentation and ultimately result in poorer model performance for this group. Overall, meaningful differences in fairness metrics highlight the necessity for continuous monitoring of algorithmic fairness. Although various methods for measuring and mitigating discrimination have been developed, no universal standard applies across ML applications (33). Prospective validation, transparent threshold selection, and sensitivity analyses that equalize TPR or FPR across groups are pragmatic next steps.

### Strengths and limitations

Our study has several strengths. It incorporated both medical and nonmedical factors in an ML model for the prediction of early onset hypertension, demonstrating that key influences span these domains. Additionally, the study evaluated model fairness across different predictors, underscoring the importance of monitoring algorithmic fairness. The importance of various features was assessed across subgroups, indicating that some factors have a greater impact on predictions among some groups. Sensitivity analyses further supported the robustness of the model’s performance and the significance of the features.

Our study also had several limitations. First, the model may have been subjected to overfitting due to the inclusion of a large number of predictors. To address this, we applied the LASSO method for feature selection, which enabled us to retain only the most relevant variables by shrinking less informative coefficients toward zero (15). Furthermore, we implemented 5-fold cross-validation to obtain a more reliable estimate of the model’s generalization performance and to minimize the risk of overfitting (17). Second, while the MEPS database is a nationally representative panel survey, it has several limitations. For example, it lacks laboratory data, such as data on systolic and diastolic blood pressure, which are relevant predictors of hypertension identified in previous research (26,28,29). Moreover, the data were self-reported, which makes them susceptible to recall bias. To mitigate this, we redefined early onset hypertension using both self-reported and diagnosis codes and observed similar results for the key predictive features. Third, some key features had missing data; in addition to the imputation approach, we conducted a sensitivity analysis restricted to people with complete data and found that the key predictive features nearly remained consistent. Lastly, because MEPS is a cross-sectional survey, following up with participants to investigate the age of early onset using this data set is difficult. To address this limitation, we are considering using a longitudinal dataset in a follow-up study to examine incident onset and temporal sequencing.

### Conclusion

We developed an interpretable ML model with strong predictive performance for early onset hypertension in US adults. Its high ROC AUC and moderate PR AUC support its use in population-level, recall-oriented screening to identify at-risk people for follow-up rather than definitive diagnosis. Both medical and nonmedical predictors contributed meaningfully; among adults older than 40 years, a diagnosis of high cholesterol was particularly influential. Observed performance differences across racial and ethnic groups highlight the need for routine fairness evaluation and model refinement. Overall, these findings support the potential of ML to enhance early detection of early onset hypertension in younger adults and enable timely preventive interventions. By integrating an array of clinical, behavioral, and contextual factors, the model provides a comprehensive risk profile to inform personalized care and guide public health strategies.
